# Acute renal failure as the initial presentation of T-lymphoblastic lymphoma: A case report 

**DOI:** 10.5414/CNCS111766

**Published:** 2025-12-19

**Authors:** Noureldien Darwish, Andrea Lightle

**Affiliations:** Department of Pathology and Laboratory Medicine, Albany Medical College, Albany NY, USA

**Keywords:** acute renal failure, hypertensive crisis, renal mass, T-cell lymphoblastic leukemia/lymphoma

## Abstract

T-lymphoblastic lymphoma/leukemia (T-LBL) is a rare and aggressive hematologic malignancy characterized by a neoplastic proliferation of immature T lymphocytes that is restricted to nodal/extra-nodal sites with minimal involvement of bone marrow. While T-LBL is the second most frequent subtype of pediatric non-Hodgkin lymphoma, primary renal involvement in T-LBL is exceedingly rare and can pose a significant diagnostic challenge. We present the case of an 11-year-old male who initially presented with new-onset seizure, hypertensive crisis, and acute renal failure. Renal ultrasounds demonstrated enlarged kidneys with loss of corticomedullary differentiation suggestive of medical renal disease. A kidney biopsy was performed, revealing an atypical interstitial T-cell infiltrate with diffuse expression of CD4, CD8, and TdT, raising concern for T-cell acute lymphoblastic lymphoma (instead of leukemia) (T-ALL/LBL). A subsequent bone marrow biopsy was negative, and no other sites of involvement were identified on PET/CT, so chemotherapy was deferred until the diagnosis could be confirmed. The patient re-presented 2 months later with visual changes and diffuse leptomeningeal enhancement on MRI. Repeat kidney biopsy with flow cytometry demonstrated a population of aberrant T cells with CD4/CD8 co-expression. A repeat bone marrow biopsy contained < 1% blasts, and no aberrant lymphocytes were detected in peripheral blood. CT scan revealed new retroperitoneal adenopathy with infiltrative disease involving kidneys, pancreas, adrenal glands, and liver, consistent with stage IV T-LBL.Kidney involvement in acute LBL is uncommon, and renal failure due to leukemic infiltration is rarely reported. This case underscores the importance of performing kidney biopsies in cases of unexplained acute renal failure and considering lymphoma in the differential for interstitial nephritis, even in the absence of abnormal hematological findings.

## Introduction 

Acute lymphoblastic leukemia (ALL) is the most common childhood cancer, primarily affecting hematopoietic tissues [1]. T-cell acute lymphoblastic leukemia (T-ALL) has an incidence of ~ 0.13 cases per 100,000 people per year in the United States, according to SEER data from 2001 to 2014 [2]. Among pediatric ALL cases, ~ 15% are of T-cell lineage [3]. 

Renal involvement occurs in ~ 7% of cases, with kidney enlargement detected via intravenous pyelography, though it is usually asymptomatic and not associated with impaired renal function [4, 5]. Routine radiologic evaluation of the kidneys is not typically performed, and nephromegaly does not have prognostic significance. Acute renal failure (ARF) in ALL is often caused by tumor lysis syndrome (TLS) following chemotherapy, marked by elevated levels of uric acid, potassium, phosphate, and creatinine [1, 6]. Clinical TLS is rare, and spontaneous tumor lysis prior to treatment is very uncommon [7]. 

Here, we describe a case of a boy with T-cell leukemia/lymphoma presenting with ARF and diffuse kidney infiltration, emphasizing the diagnostic challenges and therapeutic approach. 

## Case report 

An 11-year-old male with a history of autism spectrum disorder and no other significant medical history presented with a seizure in the setting of hypertensive crisis and ARF. His blood pressure was 200/110 mmHg with a heart rate of 130 beats per minute. Laboratory evaluation revealed a serum creatinine of 8.24 mg/dL, blood urea nitrogen (BUN) of 109 mg/dL, and uric acid of 42.9 mg/dL. He was started on a nicardipine drip, bicarbonate, and labetalol. 

The initial working diagnosis included posterior reversible encephalopathy syndrome (PRES) and autoimmune nephritis, given the severity of his neurological symptoms and the requirement for dialysis. Brain MRI revealed non-specific findings, and abdominal ultrasound showed bilateral renal enlargement (15.2 cm on the left and 14.9 cm on the right) with loss of cortico-medullary differentiation. Renal biopsy was performed, and an initial histologic impression of interstitial nephritis prompted initiation of steroid therapy. Further workup of the kidney biopsy revealed an atypical T-cell infiltrate with irregular nuclear contours, prominent nucleoli, scant cytoplasm, and expression of CD3, CD4, CD8, and TdT. The patient had no associated B symptoms (fever, weight loss, or night sweats), and staging work-up including bone marrow biopsy, cerebrospinal fluid (CSF) analysis, peripheral blood studies, and PET scan showed no evidence of systemic involvement. 

While the renal biopsy findings raised significant concern for an underlying hematologic malignancy, flow cytometry was not available to confirm the presence of a clonal T-cell population. Given the atypical presentation of isolated renal involvement without lymphadenopathy or mediastinal mass and the patient’s prior steroid treatment (which resulted in clinical improvement and may have obscured diagnostic features), the clinical team proceeded cautiously, deferring definitive lymphoma-directed therapy until a definitive diagnosis could be established. 

Due to logistical factors and clinical complications including recurrent hypertensive crises it took ~ 5 weeks to complete additional testing, including repeat bone marrow biopsy and PET/CT. The patient was readmitted for completion of this workup when he developed visual disturbances, and oncology was re-consulted at that time. 

**Figure 1.**
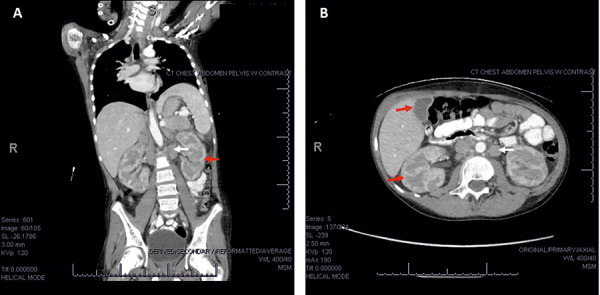
Computed tomography scan of the chest, abdomen, and pelvis from the patient’s second admission. Axial (A) and coronal (B) images show enhancing tumors primarily invading the kidneys, adrenal glands, and liver (red arrows). Retroperitoneal adenopathy is also noted (white arrows).

At this time, physical examination revealed minimal cervical lymphadenopathy more prominent on the left, without supraclavicular or axillary adenopathy. Repeat abdominal imaging and CT scan demonstrated persistent bilateral renal enlargement with new retroperitoneal adenopathy and suspicious infiltrative changes involving the pancreas, adrenal glands, and liver (Figure 1). 

**Figure 2.**
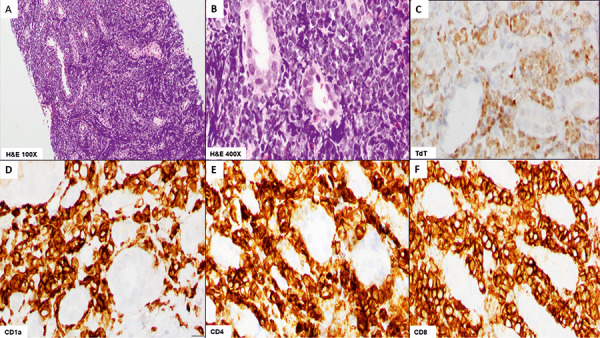
Biopsy of the right kidney. The neoplastic cells are primarily arranged in sheets around the renal tubules and exhibit nuclear atypia (A: H & E × 100, B: H & E × 400). Immunohistochemistry results show diffuse expression of TdT (C) and CD1a (D) with co-expression of CD4 (E) and CD8 (F) (all × 400).

Microscopic examination of the repeat kidney biopsy again showed diffuse infiltration by atypical lymphoid cells. An expanded immunohistochemistry panel demonstrated the malignant cells to be positive for CD1a, BCL6, CD2, CD3, CD5, CD7, CD4, CD8 (dim), CD10, and TdT, and negative for CD34, PAX5, CD20, EBER in situ hybridization, CD30, ALK1, granzyme B, PD-1, and CD56, consistent with a T-cell lymphoplastic lymphoma (Figure 2). Flow cytometry performed on the kidney biopsy confirmed 25% clonal T cells positive for CD2, dim CD3, CD4, dim CD5, CD7, CD8, and moderate CD45. Bone marrow examination and peripheral blood studies were negative for a lymphoblast population. 

The final diagnosis was T-lymphoblastic lymphoma involving the kidneys. Following diagnostic confirmation, the patient was started on appropriate treatment, including methotrexate, hydrocortisone, and cytarabine. By day 23, the patient began to show marked clinical improvement. He remains under close follow-up with our oncology team and, overall, is currently in stable and improved condition. 

## Discussion 

ALL is the most common type of hematologic malignancy in children, accounting for a significant proportion of pediatric cancers [8]. T-ALL develops when immature T cells stop maturing properly and begin to grow uncontrollably. While T-ALL makes up ~ 15% of childhood ALL cases, it accounts for ~ 25% in adults [3]. 

Renal involvement in ALL is relatively uncommon [2, 9]. While kidney enlargement is often observed through imaging techniques, it is generally asymptomatic and does not affect renal function [4, 10]. In most cases, renal dysfunction is not attributed to the leukemia itself but rather to TLS following chemotherapy. Spontaneous tumor lysis prior to treatment, leading to ARF, is exceedingly rare [6, 11]. 

Our case report highlights a unique presentation of T-cell lymphoblastic lymphoma (T-LBL) in an 11-year-old male, in which the initial manifestations were seizures, hypertensive crisis, and renal failure. A kidney biopsy revealed an atypical T-cell infiltrate, leading to a re-evaluation of the clinical diagnosis of PRES and interstitial nephritis. This patient’s renal involvement was particularly unusual, as there were no systemic symptoms commonly seen in lymphoma such as weight loss, fever, or night sweats. This underscores the importance of considering malignancy in pediatric patients with unexplained renal failure, even when classic systemic symptoms are absent. 

Upon initial presentation, the patient was treated with medications to manage his blood pressure and renal dysfunction, and he showed improvement after initiating therapy. After his discharge, he was readmitted for vision problems, and further imaging revealed infiltrative disease involving not only the kidneys but also the retroperitoneal lymph nodes, pancreas, adrenal glands, and liver. This rapid progression of disease reinforces the need for vigilant monitoring and re-evaluation in such cases. The patient was ultimately diagnosed with T-LBL, with confirmation via flow cytometry and immunohistochemical staining. 

Importantly in our case, the initial PET scan was negative despite biopsy-proven renal involvement by lymphoma. This may be attributed to the physiological excretion of FDG through the kidneys which creates high background activity and can obscure parenchymal lesions. Therefore, it is essential to note that a negative PET scan does not exclude renal involvement in cases of lymphoma. 

Interestingly, this case is part of a small but growing body of literature that describes renal involvement in T-LBL. Table 1 summarizes other reported cases of T-cell lymphoma involving the kidneys [12, 13, 14]. In a 2022 study by Nuguri et al. [12], a 50-year-old male with a history of thymoma presented with abdominal pain and progressive renal failure, and was ultimately diagnosed with high-grade T-cell lymphoma. Similarly, a 2003 study by Nizze et al. [14] reported a 13-year-old male who presented with ARF as the initial manifestation of T-cell leukemia/lymphoma. In this case, the ARF was due to kidney infiltration by T cells, with subsequent recovery following chemotherapy. Other cases in the literature also point to the diverse presentations and variable outcomes of patients with renal involvement in T-cell lymphoma or leukemia. Notably, some cases report patients who progressed to end-stage renal failure requiring dialysis, while others showed improvement with appropriate chemotherapy. 

Further studies are needed to establish more definitive diagnostic and treatment guidelines for renal involvement in T-LBL. The use of renal biopsy, in conjunction with bone marrow analysis and imaging, can help distinguish malignancy from other causes of renal failure, ensuring timely and accurate treatment. Additionally, the molecular characteristics of the disease, such as the immunophenotypic profile and flow cytometry analysis, are essential in confirming the diagnosis, as seen in this case. 

In conclusion, this case reinforces the importance of considering a broad differential diagnosis in children presenting with unexplained renal failure, particularly when there is no evidence of systemic illness. Early recognition of malignancies such as T-LBL and prompt initiation of chemotherapy can significantly improve outcomes, as demonstrated by the patient’s clinical recovery. Furthermore, it is critical to document the varied presentations of T-LBL in the literature to guide future clinicians in identifying similar cases. 

## Conclusion 

Our case report highlights a unique presentation of extra-nodal T-LBL in an 11-year-old male manifesting with seizures, hypertensive crisis, and renal failure. The initial clinical impression of PRES and interstitial nephritis led to the patient being treated with high-dose steroids, which likely masked the disease on the subsequent bone marrow biopsy and PET/CT performed during his first admission. This emphasizes the importance of including lymphoproliferative disease in the differential diagnosis of interstitial nephritis, regardless of the patient’s age. Early diagnosis through biopsy and immunohistochemical analysis is crucial for guiding appropriate treatment and improving patient outcomes. Though rare, T-cell leukemia/lymphoma should not be overlooked in cases of unexplained renal failure, as prompt treatment with chemotherapy can significantly impact prognosis. 

## Acknowledgment 

This research received no specific grant from any funding agency in the public, commercial, or not-for-profit sectors. 

## Ethical statement 

The authors are accountable for all aspects of the work in ensuring that questions related to the accuracy or integrity of any part of the work are appropriately investigated and resolved. All procedures performed in this study were in accordance with the ethical standards of the Institutional and/or national research committee(s) and with the Helsinki Declaration (as revised in 2013). Publication of this case report and accompanying images was waived from patient consent according to the Institutional Review Board at Albany Medical College (Albany, NY, USA) ethics committee/institutional review board with a waiver of patients’ consent. 

## Data availability 

Access to data is permitted with the authors’ permission. 

## Authors’ contributions 

ND and AL contributed to collecting information, writing, and editing the manuscript; supervision or mentorship: AL. AL takes responsibility that this study has been reported honestly, accurately and transparently, and accepts accountability for the overall work by ensuring that questions pertaining to the accuracy or integrity of any portion of the work are appropriately investigated and resolved.

## Funding 

This research received no specific grant from any funding agency in the public, commercial, or not-for-profit sectors. 

## Conflict of interest 

The authors have no conflict of interest to declare. 

**Table 1.** Kidney involvement with T-cell leukemia/lymphoma. The present case, along with six other cases reported in the medical literature, is summarized below.

Reference	Patient age (years)	Sex	History	Serum creatinine (mg/dL)	Diagnosis	Follow-up
Our case	11	M	Hypertensive crisis and ARF as initial presentation	8.2	T-acute lymphoblastic lymphoma.	Chemotherapy started, clinically improved after 4 months.
Nuguri et al. 2022 [12]	50	M	History of thymoma, post chemo- and radiotherapy, present with abdominal pain.	5.6	High-grade T-cell lymphoma.	Lost to follow-up.
	11	M	Rapidly progressive renal failure.	4.6	T-acute lymphoblastic lymphoma.	Died due to sepsis.
	35	M	Diagnosed case of NHL, ARF.	4.8	T-cell lymphoma.	Alive on dialysis.
Kamińska et al. 2020 [13]	34	N/A	Post-renal transplant due to glomerulonephritis.	N/A	T-cell lymphoma.	Died after 14 months.
	44	N/A	Post-renal transplant due to glomerulonephritis	N/A	T-cell lymphoma.	Chemotherapy started and cured in 12 months.
Nizze et al. 2003 [14]	13	M	ARF as initial presentation	1.84	T-acute lymphoblastic lymphoma.	Chemotherapy resulted in total recovery.

M = male; ARF = acute renal failure; NHL = non-Hodgkin lymphoma; N/A = not applicable.
